# Sleep Apnea and Paroxysmal Atrial Fibrillation: Diurnal Patterning of Autonomic Dysfunction and Influence of CPAP Therapy

**DOI:** 10.1002/joa3.70352

**Published:** 2026-06-10

**Authors:** Sepideh Khazaie, Lu Wang, Farhad Kaffashi, Ahana Sandhu, Mina K. Chung, Catherine M. Heinzinger, David R. Van Wagoner, Kenneth A. Loparo, Harneet K. Walia, James F. Bena, Reena Mehra

**Affiliations:** ^1^ Sleep Disorders Center Neurological Institute Cleveland Ohio USA; ^2^ Department of Quantitative Health Sciences Cleveland Clinic Cleveland Ohio USA; ^3^ Institute for Smart, Secure and Connected Systems: ISSACS Case Western Reserve University Cleveland Ohio USA; ^4^ Sleep Disorders Bakersfield California USA; ^5^ The Department of Cardiovascular Medicine, Heart, Vascular and Thoracic Institute Cleveland Clinic Cleveland Ohio USA; ^6^ Department of Cardiovascular and Metabolic Sciences, Lerner Research Institute Cleveland Clinic Lerner College of Medicine of Case Western Reserve University Cleveland Ohio USA; ^7^ Lerner Research Institute Cleveland Clinic Cleveland Ohio USA; ^8^ Miami Cardiac and Vascular Institute Baptist Health South Florida Miami Florida USA; ^9^ Division of Pulmonary, Critical Care and Sleep Medicine, Department of Medicine University of Washington Seattle Washington USA

**Keywords:** cardiac arrhythmias, continuous positive airway pressure, sleep apnea

## Abstract

**Objective:**

Autonomic nervous system (ANS) dysfunction is implicated in sleep‐disordered breathing (SDB) and atrial fibrillation (AF); however, diurnal patterning of ANS function in SDB is unclear. We hypothesize diurnal variation of heart rate variability (HRV) in paroxysmal AF (PAF) in SDB and alteration by continuous positive airway pressure (CPAP).

**Method:**

Data from the Sleep Apnea and Atrial Fibrillation Biomarkers and Electrophysiologic Atrial Triggers (SAFEBEAT) study including 7–24 days of electrocardiography (ECG), concomitant actigraphy and polysomnography at baseline and 3 months post‐CPAP initiation were analyzed. Linear mixed‐effects models were used to assess (1) SDB: apnea hypopnea index (AHI), hypoxia (%sleep time with SaO_2_ < 90% and nadir SaO_2_) and (2) diurnal average HRV in 2 domains: *frequency domain* (*sympathovagal*) with low‐frequency (LF) power (LFP), high‐frequency (HF) power (HFP), LF/HF ratio (LHR), and *time domain* with mean of normal R‐R interval (MNN), standard deviation of NN intervals (SDNN), Poincare plot standard deviation short‐term and long‐term variability (SD1, SD2) and effect of CPAP. All results were adjusted for age, sex, race, BMI, and use of antihypertensive, antiarrhythmic, and atrioventricular nodal blockade medications.

**Results:**

In 44 869 5‐min epochs from 109 participants with SDB and PAF, associations of AHI and LFP, nadir SaO_2_ with sympathovagal measures (LFP, LHR) and time domain measures (SDNN, SD2) were observed during wakefulness. Sleep–wake interactions were observed for multiple HRV measures, with associations generally more evident during wakefulness. From baseline to follow‐up after CPAP, wakefulness HRV measures showed an increase in MNN and decreases in SDNN, RMSSD, CV, SD1, and SD2. During sleep, MNN, SD2, and LHR increased.

**Conclusions:**

Autonomic measures exhibited diurnal variation in moderate–severe SDB and were influenced by CPAP. Association with SDB severity was more pronounced during wakefulness, providing key insights into likelihood of sustained chronobiologic electrophysiological remodeling.

## Graphical abstract

Autonomic dysfunction in sleep apnea shows diurnal variation, with stronger associations between SDB severity and HRV during wakefulness than sleep. CPAP therapy induces complex, state‐dependent changes in HRV, suggesting that daytime autonomic alterations may better reflect sustained cardiovascular risk in patients with paroxysmal atrial fibrillation.

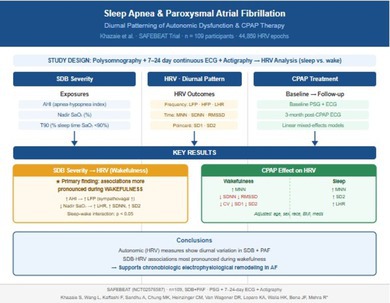

## 1 Introduction

Atrial fibrillation (AF) has been coined the epidemic of the millennium and its incidence is increasing rapidly, projected to increase 5‐fold, and afflict up to 16 million in the USA by the year 2050, with annual costs over $6.7 billion [1, 2]. A recent American Heart Association statement highlights key aspects of sleep disordered breathing (SDB) and AF inter‐relationships including an elevated SDB prevalence range of 21% to 80% in AF [3]. Both SDB and AF have been linked to alterations in autonomic nervous system (ANS) function. SDB, characterized by repeated episodes of partial or complete cessation of breathing during sleep, is associated with autonomic imbalance characterized by juxtaposition of post‐respiratory event sympathetic nervous system overactivity with preceding parasympathetic nervous system activity enhancement during the respiratory event [4]. AF is similarly associated with ANS dysfunction, with enhanced sympathetic nervous system activation, although cholinergic AF has also been well‐described [5].

ANS dysfunction is implicated in the pathophysiology of SDB‐induced atrial arrhythmogenesis. This is underscored by evidence of sleep apnea‐induced AF attenuation after pulmonary vein ganglionated plexi isolation and pharmacological neurohumoral blockade [3]. Hypoxemia in the context of apneic and hypopneic events can trigger vagal responses with synergy from stimulation of the parasympathetic nervous system by increased negative intrathoracic pressure induced by occlusion of the upper airway [6]. Overall, SDB and AF are both associated with ANS dysfunction, which may offer a promising avenue for the management of these conditions [7, 8].

SDB severity can vary night‐to‐night in a single patient in part due to sleep state and positional variability [9, 10]. Nights with the highest SDB burden are associated with a more than 2‐fold higher risk of at least 1 h of AF than nights with the lowest SDB burden [11]. While these results are highly informative, the role of hypoxia is unclear as it was not measured in prior studies designed to examine the temporality of respiratory events in SDB and AF paroxysms. Existing data are also limited by non‐standard methods to measure both apnea and hypopneas [9, 10, 11]. Novel technological advances have the potential to change the landscape of widespread screening and management of SDB, particularly with respect to mobile health technologies that integrate electrocardiogram (ECG) and autonomic measures improving our ability to examine multi‐day and night sleep and electrophysiologic data [12].

Our goal, therefore, is to extend existing knowledge of the temporality of SDB and arrhythmic events by now investigating diurnal fluctuations in ANS biomarkers in relation to SDB exposures. We leverage a unique data set of synchronous collection of continuous ECG and multi‐day and night actigraphy data collection over weeks, along with overlapping overnight polysomnography in a well‐phenotyped cohort of participants with paroxysmal AF (PAF) and SDB, thereby allowing for temporal and integrative analyses.

Using heart rate variability (HRV) parameters as representative biomarkers of ANS function, we hypothesize that (1) there will be diurnal differences between PAF severity and SDB severity such that the nocturnal association of SDB and HRV indices will be greater than the daytime association, and (2) in those with PAF, SDB treatment with continuous positive airway pressure (CPAP) will alter HRV. To our knowledge, this is the first study to leverage continuous multi‐day ECG and actigraphy data alongside polysomnography to comprehensively examine diurnal variations in HRV in relation to sleep‐disordered breathing severity in individuals with paroxysmal atrial fibrillation. Additionally, our analysis uniquely evaluates the impact of CPAP therapy on these autonomic patterns, which have not been previously described. Clinical relevance resides in the ability to better understand ANS‐specific chronobiology in SDB and AF, thus potentially informing clinical recommendations such as chronotherapeutic approaches.

## 2 Methods

### 2.1 Setting and Participants, Study Design

Over the period March 2012 to March 2017, patients with PAF, defined as recurrent episodes that self‐terminate within 7 days, were enrolled in the NHLBI/NIH Sleep Apnea and Atrial Fibrillation Biomarkers and Electrophysiologic Atrial Triggers trial (SAFEBEAT, NCT02576587). Sleep studies were conducted in the Clinical Research Units of University Hospitals of Cleveland or Cleveland Clinic. The University Hospitals Case Medical Center IRB and Cleveland Clinic IRB each approved this study (IRB approval number: 13‐594), and informed consent was obtained for all participants. Exclusion criteria have been previously reported [4].

Overnight polysomnogram data was collected at the Clinical Research Unit. Data collected include demographic information, medication use, and medical history data via questionnaires. Body mass index (BMI; kg/m^2^) was calculated based on weight measurements taken with standard balance beams or calibrated digital scales and height measurements taken using a wall‐mounted Harpenden stadiometer. Cardiovascular disease was defined by a history of myocardial infarction, coronary angioplasty, stroke, and/or coronary artery bypass graft surgery self‐reported by the participant and verified by the electronic medical record.

### 2.2 Data Collection

#### 2.2.1 Attended Polysomnography

The Compumedics E‐series data acquisition system was used for polysomnography (Abbottsford, AU) including cortical electroencephalograms (EEG), bilateral electro‐oculograms, a bipolar submental electromyogram, thoracic and abdominal respiratory inductance plethysmography, airflow (by nasal‐oral thermocouple and nasal cannula pressure recording), oximetry (using highly sensitive finger pulse oximeter, sampling frequency 25 Hz), ECG at 250 Hz (used to derive HRV measures of autonomic function); body position (mercury switch sensor), and bilateral leg movements. Using standardized techniques, a certified technician calibrated sensors and checked signal quality/impedance. Central and obstructive apneas were distinguished based on the absence or presence of respiratory effort, respectively. Hypopneas were scored as a 50% amplitude reduction in inductance or flow associated with 3% oxygen desaturation or EEG cortical arousal. After at least 10 continuous seconds of sleep, arousal was defined as a sudden shift in EEG frequency to wake activity lasting at least 3 s [13]. Over the entire sleep period, the number of apneas and hypopneas were used to calculate the apnea hypopnea index (AHI). Sleep‐related hypoxia was defined by the percentage of sleep time with oxygen saturation (SaO_2_) less than 90% (T90) and the nadir SaO_2_. A standardized protocol was used to train the research staff who conducted the study and a certified polysomnologist, blind to clinical information, scored the sleep studies [14, 15]. All scored measures had greater than 95% inter‐scorer reliability [4].

#### 2.2.2 Continuous ECG Monitoring

An ECG monitoring device with a sampling rate of 250 Hz (Heartrak External Cardiac Ambulatory Telemetry) was used over a 1‐week period in patients with PAF following the baseline research visit and in a subset of patients treated with CPAP at a 3‐month follow‐up visit. On the morning following the sleep study, a trained technician applied the ECG sensors. Participants were instructed to engage in normal activities, remove their monitors before bathing, and reapply the sensors after bathing.

#### 2.2.3 Actigraphy

An accelerometer (GT3X+, Actigraph Co., Ft Walton Beach, Fl) was used for 7–24 days to measure the duration and intensity of physical activity and sleep, simultaneously with continuous ECG monitoring. Participants were instructed to use the actigraph device throughout the day, except while in water. This device provides objective measurements of sleep duration and activity during sleep by recording temperature, ambient light, and activity during sleep. Wakefulness and sleep were ascertained from the objective actigraph‐based measures using the validated Cole‐Kripke algorithm, which primarily distinguishes primary sleep periods but may not fully account for daytime naps or fragmented sleep, potentially influencing autonomic function assessments.

#### 2.2.4 Electrophysiological Signal Processing Analyses

ECG measures were analyzed as continuous outcomes, averaging information from 5‐min intervals of normal sinus rhythm periods, thus deriving an aggregate summary of electrophysiological variability. To account for sleep state, 5‐min ECG windows during sleep versus wake were analyzed and concatenated 30‐s epochs were used in the analysis.

HRV signal processing analyses were performed using the lead II ECG signal (sampled at 250 Hz) of continuous ECG monitoring. ECG data were pre‐processed using sequentially automated and manual scoring using Somte software (Compumedics Inc., Melbourne, Australia). Automated analyses were applied excluding segments of atrial and ventricular ectopic beats. During the analysis, all 5‐min segments (standard duration to ensure stationarity of the ECG time series) of the continuous ECG recordings without ectopy or artifact as determined by pre‐processed ECG data were included. Artifact rejection algorithms excluded segments of heart rate lower than 30 beats/min or greater than 180 beats/min or instantaneous heart rate changes exceeding 80 beats/min between consecutive R‐R intervals.

Frequency‐based HRV measures were analyzed using approaches for non‐uniformly distributed R‐R interval data. Spectral analysis was performed using Fast Fourier transform–based methods to decompose heart rate time series into frequency components. This approach was used to calculate the normalized low‐frequency power (LFP) (from 0.04 to 0.15 Hz) and the normalized high‐frequency power (HF) (from 0.15 to 0.4 Hz). In the HF range, parasympathetic activity of the sinoatrial node is primarily responsible, while in the LF range, sympathetic activity is predominant. The LF/HF ratio reflects sympathovagal balance.

Time‐domain HRV measures were derived from the normal‐normal (beat) intervals (NN), or from Poincare' plots, that is, using normal‐normal intervals (NN) determined by sinus node depolarizations detected between adjacent QRS complexes, mean and standard deviation of the time intervals between normal beats (MNN, SDNN), root mean square differences between adjacent normal beats (RMSSD), coefficient of variation (CV) of the R‐R interval time series, and standard deviation of the minor and major axis (SD1, SD2) and their ratio (SD Ratio). Finally, non‐linear measurements, that is, detrended fluctuation analysis (DFA), allowed quantification of the unpredictability of the R‐R time series.

HRV analyses were performed by researchers blinded to participants' clinical characteristics and CPAP status.

#### 2.2.5 CPAP Protocol for Participants With SDB and PAF

Participants with moderate to severe SDB (AHI > 15), but without evidence of central apnea (central apnea index > 5) or Cheyne Stokes Respirations via baseline polysomnogram, and who have PAF, underwent a 5–7 day home‐based titration with an auto‐titrating CPAP machine (Respironics Autopap System One with humidifier) to identify the optimal CPAP setting, with pressures ranging from 4 to 20 cm H_2_O. Participants were educated on the use of CPAP and mask fit was optimized. An in‐person visit was scheduled for those participants with non‐adherence due to mask fit or leak noted on more than two consecutive nights. An overnight visit was scheduled after 3 months of wearing CPAP, during which the same measures performed at the baseline visit were collected. An overnight visit was scheduled after 3 months of wearing CPAP, during which the same measures performed at the baseline visit were collected. Objective CPAP adherence data, defined as ≥ 4 h of use on ≥ 70% of nights, was available in a subset of participants and incorporated as a covariate in statistical models. The 3‐month follow‐up duration was selected based on prior studies demonstrating detectable changes in autonomic function and cardiovascular parameters following CPAP therapy within this timeframe, though longer periods may be required to fully capture autonomic remodeling.

### 2.3 Statistical Analyses

Demographic and baseline sleep indices were described using frequencies and percentages for categorical factors, while continuous measures were described with means and standard deviations or medians and quartiles based on distribution. Comparisons by PAF occurrence during continuous ECG were performed using two‐sample *t*‐test or Wilcoxon rank sum test for continuous measures, and Pearson chi‐square tests or Fisher exact tests for categorical factors.

To account for the correlation of repeated epochs in the same patient, a linear mixed‐effects model (LMM) assuming a compound symmetry correlation structure was utilized. This model summarized HRV measures by sleep and wake states at both baseline and follow‐up. Interaction terms between sleep status and SDB indices were included to formally test whether associations (i.e., slopes) differed between sleep and wake conditions. Additionally, it assessed the relationship of baseline HRV measures with baseline apnea‐hypopnea index (AHI) (per 5‐unit increase), hypoxia (T90), and nadir SaO_2_ (per 5% increase). Given the skewed distribution of hypoxia (T90), we dichotomized this variable at the median for our analysis. Measures with a skewed distribution (CV, SD1, SD2, SD Ratio, LFP, HFP, and LHR) underwent logarithmic transformation to normalize data and meet model assumptions, as determined by visual inspection of histograms and skewness statistics (> 1 or < −1), and were transformed back for presentation. The statistical interaction of sleep and wake states in relation to sleep and HRV measures was also analyzed to assess diurnal differences in these associations. An overall significance level of 0.05 was applied to all analyses, and SAS software (version 9.4, Cary, NC) was used. Given the exploratory, hypothesis‐generating nature of this secondary analysis, adjustment for multiple comparisons was not applied in order to avoid overlooking potentially informative associations that may warrant future investigation. All multivariable models were adjusted for age, sex, race, BMI, and use of antihypertensive, antiarrhythmic, and atrioventricular nodal blockade medications. A priori power analysis was not conducted as this was a secondary analysis of available SAFEBEAT trial data.

## 3 Results

### 3.1 Cohort Description

Table 1 reports the baseline characteristics of the cohort. The 109 participants in the overall cohort were 60.3 ± 11.8 years old and 99.1% white with a BMI of 32.2 ± 6.6 kg/m^2^ and AHI of 13.4 (IQR 4.0–27.9). The analytic sample comprised 44 869 30‐s epochs (artifact‐free and without ectopy or AF paroxysms) from 109 participants with a diagnosis of PAF, among which 79 patients had both baseline and follow‐up and 3 patients had only follow‐up data. Continuous ECG monitoring at the baseline visit had a mean duration of 9.68 ± 4.32 days (range 2–21 days) (Table 1). The median number of 5‐min ECG epochs from continuous ECG monitoring available per day at baseline was 35 (IQR 17–59) and at the follow‐up visit was 38.5 (IQR 17.5–70).

**TABLE 1:** Characteristics of participants with moderate to severe sleep disordered breathing stratified by paroxysmal atrial fibrillation.

Factor	Overall (*N* = 109)		No paroxysmal atrial fibrillation detected on ECG monitoring (*N* = 78)		Paroxysmal atrial fibrillation detected on ECG monitoring (*N* = 31)		*p*
	*N*	Statistics	*N*	Statistics	*N*	Statistics	
Age (years), mean (SD)	109	60.3 ± 11.8	78	60.2 ± 12.0	31	60.7 ± 11.7	0.83^a^
Body mass index (kg/m^2^), mean (SD)	109	32.2 ± 6.6	78	31.6 ± 6.3	31	33.7 ± 7.3	0.14^a^
Gender, *N* (%)	109		78		31		0.93^c^
Female		38 (34.9)		27 (34.6)		11 (35.5)	
Male		71 (65.1)		51 (65.4)		20 (64.5)	
Race							
White	92	92 (84.4)	78	69 (88.5)	31	23 (74.2)	0.081^d^
Asian	2	2 (1.8)	78	2 (2.6)	31	0 (0.00)	0.99^d^
Black or African American	15	15 (13.8)	78	7 (9.0)	31	8 (25.8)	**0.031** ^d^
Native Hawaiian or Other Pacific Islander	2	2 (1.8)	78	2 (2.6)	31	0 (0.00)	0.99^d^
Ethnicity	109		78		31		0.28^d^
Hispanic or Latino/a		1 (0.92)		0 (0.00)		1 (3.2)	
NOT Hispanic or Latino/a		108 (99.1)		78 (100.0)		30 (96.8)	
Apnea hypopnea index	109	13.4 [4.0, 27.9]	78	12.8 [3.7, 26.2]	31	15.0 [4.4, 30.8]	0.78^b^
% sleep time with < 90% SaO_2_ (%)	109	0.51 [0.00, 3.5]	78	0.64 [0.00, 3.5]	31	0.43 [0.00, 2.9]	0.75^b^
Nadir SaO_2_ in sleep (%)	109	86.0 [80.0, 90.0]	78	86.0 [79.0, 90.0]	31	86.0 [81.0, 90.0]	0.99^b^
Total sleep time (minutes)	109	335.0 [284.0, 373.0]	78	334.5 [277.0, 373.0]	31	336.0 [312.0, 377.0]	0.30^b^
Sleep efficiency (%)	109	73.8 [62.5, 82.7]	78	73.7 [61.8, 83.1]	31	75.0 [63.4, 82.0]	0.93^b^
Arousal index	109	19.2 [14.3, 28.4]	78	19.5 [14.3, 29.1]	31	18.6 [12.5, 25.8]	0.48^b^
Obstructive Apnea index	109	0.57 [0.00, 4.3]	78	0.75 [0.12, 5.0]	31	0.53 [0.00, 3.3]	0.35^b^
Central Apnea index	109	0.00 [0.00, 0.35]	78	0.00 [0.00, 0.44]	31	0.00 [0.00, 0.33]	0.47^b^
Time in stage 1 sleep (minutes)	109	23.0 [16.0, 32.0]	78	23.0 [16.0, 29.0]	31	27.0 [13.0, 35.0]	0.75^b^
Time in stage 2 sleep (minutes)	109	183.0 [155.0, 224.0]	78	183.0 [154.0, 220.0]	31	194.0 [167.0, 226.0]	0.38^b^
Time in stage 3–4 sleep (minutes)	109	58.0 [37.0, 77.0]	78	58.0 [37.0, 78.0]	31	56.0 [31.0, 77.0]	0.78^b^
Time in REM sleep (minutes)	109	57.0 [39.0, 72.0]	78	54.0 [33.0, 71.0]	31	64.0 [47.0, 74.0]	**0.038** ^b^
Sleep time in stage 1 sleep (%)	109	6.9 [4.1, 9.9]	78	6.9 [4.2, 10.3]	31	6.7 [3.7, 8.9]	0.66^b^
Sleep time in stage 2 sleep (%)	109	57.7 [52.1, 63.3]	78	57.8 [52.6, 63.2]	31	57.4 [50.5, 63.9]	0.99^b^
Sleep time in stage 3–4 sleep (%)	109	17.6 [11.5, 23.3]	78	17.9 [12.4, 23.9]	31	16.1 [8.4, 21.5]	0.32^b^
Sleep time in REM sleep (%)	109	16.8 [13.0, 20.3]	78	16.2 [12.2, 20.1]	31	17.9 [15.0, 21.0]	0.064^b^
LAVI	96	29.8[23.0, 35.7]	70	28.1[22.6, 33.3]	26	34.1[27.7, 41.4]	0.042^b^
LA Volume	97	62.9 [50.4, 76.1]	70	58.5 [48.0, 74.6]	27	67.2 [52.9, 84.6]	**0.048** ^b^
LA strain A4C	85	35.2 [27.7, 41.8]	64	36.9 [28.5, 42.7]	21	30.6 [24.5, 36.1]	**0.034** ^b^
LA strain A2C	84	35.1 [29.3, 40.7]	63	36.1 [31.8, 42.3]	21	33.1 [27.3, 36.6]	0.053^b^
Antihypertensives, *N* (%)	43	43 (39.4)	78	27 (34.6)	31	16 (51.6)	0.10^c^
Antiarrhythmics, *N* (%)	39	39 (35.8)	78	26 (33.3)	31	13 (41.9)	0.40^c^
Atrioventricular_nodal_blockers, *N* (%)	74	74 (67.9)	78	53 (67.9)	31	21 (67.7)	0.98^c^
Alcohol use, *N* (%)	109	69 (63.3)	78	51 (65.4)	31	18 (58.1)	0.47^c^
High blood pressure or hypertension, *N* (%)	109	64 (58.7)	78	46 (59.0)	31	18 (58.1)	0.93^c^
Diabetes, *N* (%)	109	14 (12.8)	78	9 (11.5)	31	5 (16.1)	0.53^d^
Heart attack, *N* (%)	109	3 (2.8)	78	2 (2.6)	31	1 (3.2)	0.99^d^
Stroke, *N* (%)	109	5 (4.6)	78	11 (14.1)	31	5 (16.1)	0.77^d^

*Note:* Statistics presented as Mean ± SD, Median [P25, P75], *N* (column %). All participants had a clinical diagnosis of PAF, however, some had AF observed on ECG monitoring and some participants did not have AF observed on ECG monitoring. This table stratified based upon presence or absence of observed AF on ECG monitoring. *p*‐values: a = *t*‐test, b = Wilcoxon Rank Sum test, c = Pearson's chi‐square test, d = Fisher's Exact test. Racial categories reflect self‐reported data, with some participants identifying with multiple races, leading to totals exceeding 100%. The bolded values are *p*‐values which denotes signficance.

### 3.2 Diurnal Heart Rate Variability Measures and Sleep Disordered Breathing

Table 2 shows the association between baseline HRV and sleep indices by sleep and wake, using daily average data. To enhance clarity, we prioritize clinically relevant HRV metrics (e.g., SDNN, SD2, LHR) reflecting autonomic balance and variability, with full results detailed in Table 2.

**TABLE 2:** Heart rate variability indices by sleep and wakefulness.

Variable	Sleep		Wake		*P* of interaction
	Coefficient (95% CI)	*p*	Coefficient (95% CI)	*p*	
Association with AHI (per 5 event/h)					
Time Domain Indices					
MNN	−0.0069 (−0.0140, 0.0001)	0.054	−0.0087 (−0.0157, −0.0016)	**0.017**	0.078
SDNN	−0.0004 (−0.0010, 0.0002)	0.15	−0.0009 (−0.0015, −0.0003)	**0.003**	< **0.001**
RMSSD	−0.0002 (−0.0011, 0.0007)	0.65	−0.0005 (−0.0014, 0.0004)	0.25	**0.004**
CV	−0.0100 (−0.0241, 0.0044)	0.17	−0.0209 (−0.0349, −0.0066)	**0.005**	< **0.001**
SD1	−0.0160 (−0.0417, 0.0104)	0.23	−0.0349 (−0.0601, −0.0090)	**0.009**	< **0.001**
SD2	−0.0187 (−0.0351, −0.0021)	**0.028**	−0.0310 (−0.0472, −0.0145)	< **0.001**	< **0.001**
SDRatio	0.0015 (−0.0165, 0.0199)	0.87	−0.0027 (−0.0207, 0.0156)	0.77	0.25
Frequency Domain Indices					
LFP	−0.0014 (−0.0196, 0.0171)	0.88	0.0039 (−0.0145, 0.0226)	0.68	0.16
HFP	0.0006 (−0.0261, 0.0281)	0.96	−0.0066 (−0.0332, 0.0207)	0.63	0.19
LHR	−0.0028 (−0.0419, 0.0380)	0.89	0.0118 (−0.0280, 0.0532)	0.56	**0.050**
DFA_Alpha1	0.0055 (−0.0019, 0.0128)	0.14	0.0056 (−0.0018, 0.0129)	0.14	0.95
DFA_Alpha2	0.0060 (0.0012, 0.0109)	**0.016**	0.0070 (0.0021, 0.0118)	**0.005**	0.62
Association with %sleep time with < 90% SaO_2_ (per 5%)					
Time Domain Indices					
MNN	−0.0024 (−0.0133, 0.0086)	0.67	−0.0060 (−0.0169, 0.0050)	0.28	**0.043**
SDNN	−0.0004 (−0.0013, 0.0005)	0.36	−0.0009 (−0.0018, −0.0000)	**0.047**	**0.007**
RMSSD	−0.0002 (−0.0016, 0.0011)	0.72	−0.0007 (−0.0020, 0.0006)	0.28	**0.017**
CV	−0.0127 (−0.0343, 0.0094)	0.26	−0.0215 (−0.0427, 0.0003)	0.053	0.094
SD1	−0.0099 (−0.0494, 0.0312)	0.63	−0.0326 (−0.0710, 0.0074)	0.11	**0.003**
SD2	−0.0143 (−0.0401, 0.0122)	0.29	−0.0273 (−0.0526, −0.0013)	**0.040**	**0.020**
SDRatio	0.0033 (−0.0241, 0.0315)	0.81	−0.0036 (−0.0305, 0.0242)	0.80	0.32
Frequency Domain Indices					
LFP	0.0025 (−0.0252, 0.0310)	0.86	−0.0019 (−0.0292, 0.0263)	0.89	0.53
HFP	0.0091 (−0.0317, 0.0516)	0.66	−0.0208 (−0.0601, 0.0201)	0.31	**0.003**
LHR	−0.0074 (−0.0660, 0.0548)	0.81	0.0219 (−0.0380, 0.0855)	0.48	**0.034**
DFA_Alpha1	0.0055 (−0.0058, 0.0167)	0.34	0.0089 (−0.0023, 0.0201)	0.12	0.21
DFA_Alpha2	−0.0015 (−0.0096, 0.0066)	0.71	0.0025 (−0.0053, 0.0104)	0.52	0.26
Association with Nadir SaO_2_ (per 5%)					
Time Domain Indices					
MNN	0.0161 (0.0000, 0.0323)	**0.050**	0.0152 (−0.0009, 0.0313)	0.064	0.68
SDNN	0.0006 (−0.0008, 0.0019)	0.42	0.0023 (0.0009, 0.0036)	**0.001**	< **0.001**
RMSSD	0.0008 (−0.0012, 0.0028)	0.44	0.0008 (−0.0011, 0.0028)	0.40	0.77
CV	0.0145 (−0.0180, 0.0481)	0.38	0.0659 (0.0317, 0.1011)	< **0.001**	< **0.001**
SD1	0.0594 (−0.0018, 0.1244)	0.057	0.0795 (0.0172, 0.1456)	**0.012**	0.067
SD2	0.0287 (−0.0099, 0.0688)	0.15	0.0907 (0.0499, 0.1332)	< **0.001**	< **0.001**
SDRatio	0.0313 (−0.0100, 0.0743)	0.14	−0.0090 (−0.0486, 0.0322)	0.66	< **0.001**
Frequency Domain Indices					
LFP	0.0154 (−0.0257, 0.0583)	0.46	0.0252 (−0.0162, 0.0684)	0.23	0.29
HFP	0.0403 (−0.0214, 0.1059)	0.20	0.0091 (−0.0506, 0.0725)	0.77	**0.022**
LHR	−0.0371 (−0.1203, 0.0539)	0.41	0.0135 (−0.0739, 0.1091)	0.77	**0.004**
DFA_Alpha1	−0.0202 (−0.0367, −0.0036)	**0.018**	−0.0075 (−0.0240, 0.0090)	0.37	< **0.001**
DFA_Alpha2	−0.0145 (−0.0260, −0.0030)	**0.014**	−0.0057 (−0.0170, 0.0057)	0.32	0.057

*Note:* All results adjusted for age, sex, race, BMI, and medications (would list out the 3 med categories).

Abbreviations: CV, coefficient of variation; DFA_Alpha1, short‐term scaling exponent; DFA_Alpha2, long‐term scaling exponent; HFP, power in the high frequency range (0.15–0.4 Hz); LFP, power in the low frequency range (0.04–0.15 Hz); LHR, ratio of low frequency power to high frequency power; MNN, mean NN interval; RMSSD, root mean square of successive differences; SD1, standard deviation of short‐term heart rate variability; SD2, standard deviation of long‐term heart rate variability; SDNN, standard deviation of NN intervals; SDRatio, ratio of SD1 to SD2. The bolded values are *p*‐values which denotes signficance.

**FIGURE 1:**
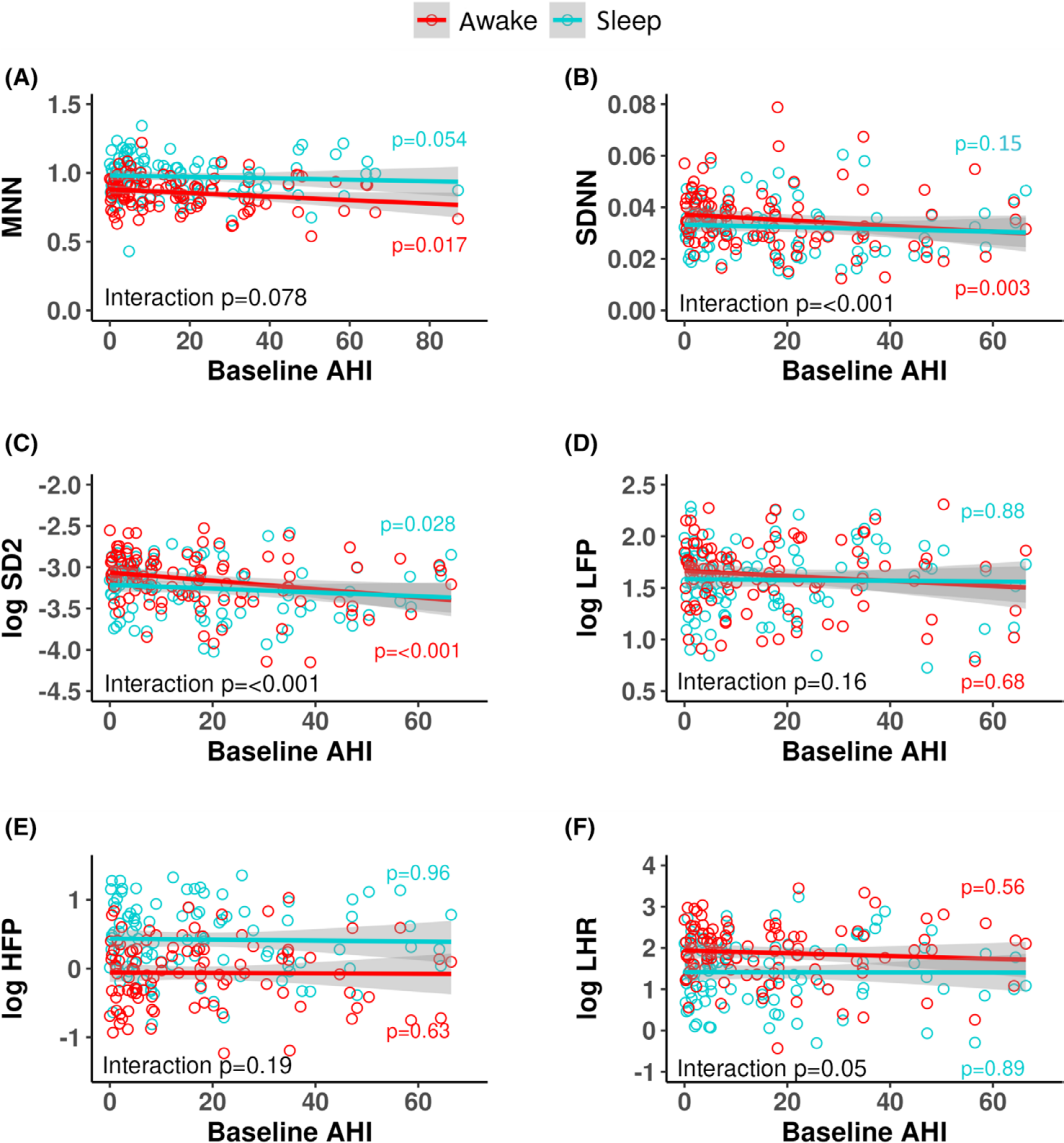
Diurnal association of sleep disordered breathing severity defined by Apnea‐Hypopnea Index (AHI) and heart rate variability measures. Estimated changes in heart rate variability measures per 5 unit increase in AHI are shown for sleep (green) and wake (red) periods. Measures shown are: (A) Mean NN interval (MNN), (B) Standard deviation of NN intervals (SDNN), (C) Log standard deviation of long‐term variability (Log SD2), (D) Log low frequency power (Log LFP), (E) Log high frequency power (Log HFP), and (F) Log low frequency/high frequency ratio (Log LHR). *p*‐values for sleep–wake interaction are displayed.

For time domain measures, SD2 had a significant negative association with AHI during both sleep (*p* = 0.028) and wake (*p* < 0.001), and the magnitude of the associations differed, with a stronger association during wakefulness than sleep (*p*‐value for interaction < 0.001). RMSSD was not associated with AHI during sleep (*p* = 0.65) or wake (*p* = 0.25); however, the relationships differed between sleep and wake (*p*‐value for interaction = 0.004). During wakefulness only, SDNN showed a significant negative association with AHI (*p* = 0.003), CV showed a significant negative association with AHI (*p* = 0.005), and SD1 showed a significant negative association with AHI (*p* = 0.009); these associations differed significantly from those observed during sleep (*p*‐value for interaction < 0.001) (Figure 1).

In analysis of frequency domain measures, LHR was not associated with AHI during sleep (*p* = 0.89) or wake (*p* = 0.56), but the sleep–wake associations differed (*p*‐value interaction = 0.050). DFA alpha2, an index of long‐term heart rate variability, had a significant positive association during sleep (*p* = 0.016) and wake (*p* = 0.005), but the sleep–wake statistical interaction was not significant (*p*‐value interaction = 0.62).

### 3.3 Diurnal Heart Rate Variability Measures and Sleep‐Related Hypoxia

**FIGURE 2:**
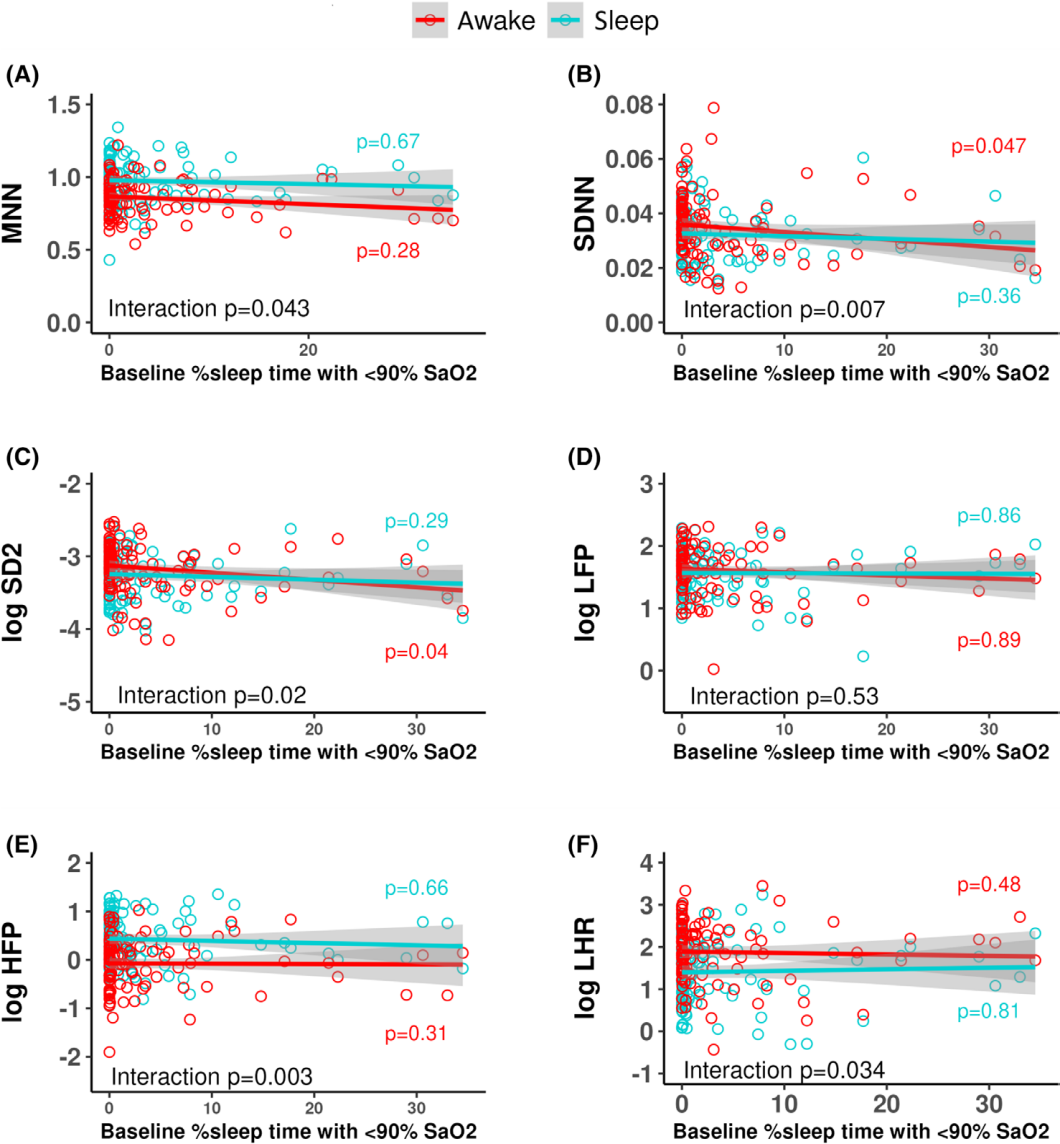
Diurnal association of sleep‐related hypoxia and heart rate variability measures. Estimated changes in heart rate variability measures per 5% increase in %TST < 90 are shown for sleep (green) and wake (red) periods. Measures shown are: (A) Mean NN interval (MNN), (B) Standard deviation of NN intervals (SDNN), (C) Log standard deviation of long‐term variability (Log SD2), (D) Log low frequency power (Log LFP), (E) Log high frequency power (Log HFP), and (F) Log low frequency/high frequency ratio (Log LHR). *p*‐values for sleep–wake interaction are displayed.

When considering sleep‐related hypoxia, time domain measures SDNN and SD2 showed significant negative associations with T90 during wakefulness only [(*p* = 0.047) and (*p* = 0.040), respectively], which differed compared to sleep (*p*‐value interaction = 0.007 and 0.020, respectively). Additionally, the interactions between T90 and the time domain and non‐linear HRV measures MNN, RMSSD, SD1, HFP and LHR were stronger during wakefulness compared to sleep with the following reported statistical interactions (MNN interaction *p* = 0.043; RMSSD interaction *p* = 0.017; SD1 interaction *p* = 0.003; HFP interaction *p* = 0.034; LHR interaction *p* = 0.001) (Figure 2).

A significant association of nadir SaO_2_ was observed with time domain measures during wakefulness [SDNN, CV and SD2] and the magnitude of association differed compared to sleep (*p*‐value interaction < 0.001). SD1 had a statistically significant positive relationship with nadir SaO_2_ in wake (*p* = 0.012), but the interaction was not significant (*p* = 0.067).

Time domain measures MNN, DFA alpha1 and DFA alpha2 had significant positive relationships with nadir SaO_2_ during sleep (*p* ≤ 0.050 for all), but only DFA alpha1 had a significant sleep–wake interaction (*p*‐value interaction < 0.001).

Frequency domain measures HFP and LHR did not have significant associations with nadir SaO_2_ during sleep or wakefulness. However, the relationships between nadir SaO_2_ and these measures differed between sleep and wake (HFP *p*‐value for interaction = 0.022; LHR *p*‐value for interaction = 0.004).

### 3.4 Effect of Continuous Positive Airway Pressure Therapy on Heart Rate Variability Measures

**FIGURE 3:**
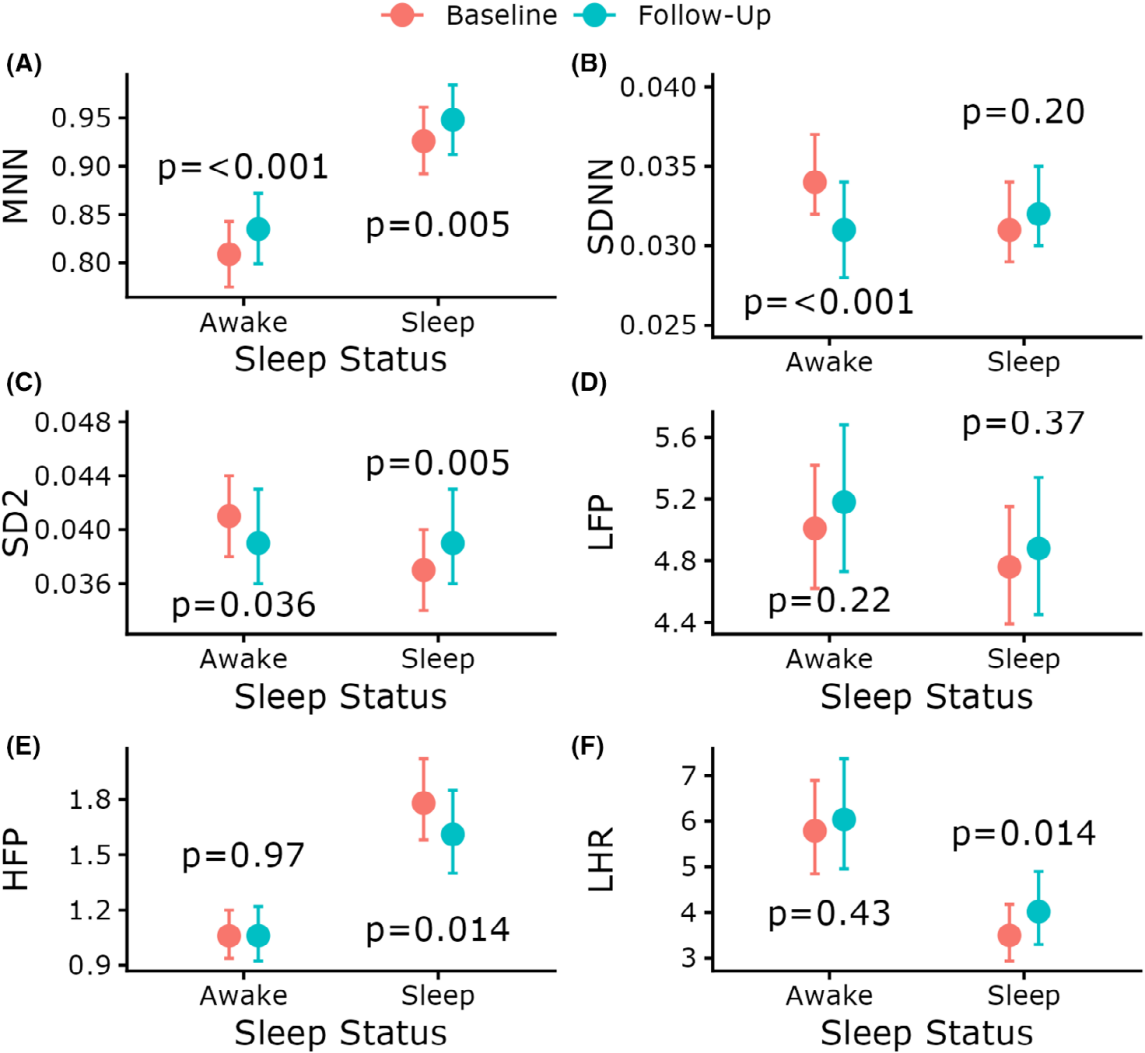
Continuous ECG monitoring‐based diurnal heart rate variability measures at baseline and 3‐month continuous positive airway pressure (CPAP) therapy follow‐up. Measures shown are: (A) Mean NN interval (MNN), (B) Standard deviation of NN intervals (SDNN), (C) Standard deviation of long‐term variability (SD2), (D) Low frequency power (LFP), (E) High frequency power (HFP), and (F) Low frequency/high frequency ratio (LHR). Baseline values are shown in red, follow‐up values in green. *p*‐values indicate change from baseline to follow‐up.

Table 3 presents least square means of HRV measures at baseline and post‐CPAP at 3‐month follow‐up by sleep/wake using daily average data. Of the 109 patients with moderate to severe SDB, 40 used CPAP with an average usage of 4.0 h (SD 2.4) per day and an average usage of 4.9 h (SD 2.0) per day per device. The median follow‐up AHI for the 35 patients with available data was 4.6 (IQR 1.8–9.1), indicating optimal control of SDB (Figure 3).

**TABLE 3:** Changes in heart rate variability measures in response to continuous positive airway pressure therapy.

Variable	Sleep			Awake		
	Baseline	Follow‐up	*p*	Baseline	Follow‐up	*p*
	LSM^a^ (95% CI)	LSM (95% CI)		LSM (95% CI)	LSM (95% CI)	
Time Domain Indices						
MNN	0.926 (0.892, 0.961)	0.948 (0.912, 0.984)	**0.005**	0.809 (0.775, 0.843)	0.835 (0.799, 0.872)	< **0.001**
SDNN	0.031 (0.029, 0.034)	0.032 (0.030, 0.035)	0.20	0.034 (0.032, 0.037)	0.031 (0.028, 0.034)	< **0.001**
RMSSD	0.027 (0.024, 0.030)	0.026 (0.022, 0.029)	0.14	0.024 (0.021, 0.028)	0.022 (0.018, 0.025)	**0.002**
CV	0.032 (0.030, 0.034)	0.033 (0.031, 0.036)	0.21	0.040 (0.038, 0.043)	0.037 (0.034, 0.040)	< **0.001**
SD1	0.017 (0.015, 0.019)	0.017 (0.015, 0.019)	0.63	0.015 (0.013, 0.016)	0.014 (0.012, 0.016)	**0.047**
SD2	0.037 (0.034, 0.040)	0.039 (0.036, 0.043)	**0.005**	0.041 (0.038, 0.044)	0.039 (0.036, 0.043)	**0.036**
SDRatio	0.471 (0.434, 0.510)	0.450 (0.411, 0.494)	0.11	0.373 (0.344, 0.404)	0.367 (0.335, 0.402)	0.59
Frequency Domain Indices						
LFP	4.76 (4.39, 5.15)	4.88 (4.45, 5.34)	0.37	5.01 (4.62, 5.42)	5.18 (4.73, 5.68)	0.22
HFP	1.78 (1.58, 2.02)	1.61 (1.40, 1.85)	**0.014**	1.060 (0.938, 1.20)	1.061 (0.924, 1.22)	0.97
LHR	3.50 (2.94, 4.18)	4.02 (3.30, 4.90)	**0.014**	5.79 (4.85, 6.90)	6.04 (4.96, 7.37)	0.43
DFA_Alpha1	0.966 (0.934, 0.997)	0.982 (0.946, 1.018)	0.13	1.053 (1.022, 1.085)	1.063 (1.027, 1.100)	0.35
DFA_Alpha2	0.647 (0.623, 0.672)	0.644 (0.611, 0.677)	0.81	0.836 (0.812, 0.860)	0.811 (0.778, 0.843)	0.06

*Note:* All results adjusted for age, sex, race, BMI, and medications (antihypertensive, antiarrhythmic and AV nodal blockers medication use). The bolded values are *p*‐values which denotes signficance.

^a^ Least squares means.

Of the time domain measures, MNN showed a statistically significant increase at 3‐month follow‐up in sleep, from 0.926 (95% CI 0.892–0.961) at baseline to 0.948 (95% CI 0.912–0.984) at follow‐up (*p* = 0.005), and in awake, from 0.809 (95% CI 0.775–0.843) at baseline to 0.835 (95% CI 0.799–0.872) at follow‐up (*p* < 0.001). SD2 significantly increased during sleep from 0.037 (95% CI 0.034–0.040) at baseline to 0.039 (95% CI 0.036–0.043) at follow‐up (*p* = 0.005), but significantly decreased during wakefulness from 0.041 (95% CI 0.038–0.044) at baseline to 0.039 (95% CI 0.036–0.043) at follow‐up (*p* = 0.036). The time domain measure, SDNN, had a significant decrease during wakefulness after CPAP therapy (*p* < 0.001); RMSSD had a significant decrease during wakefulness after CPAP therapy (*p* = 0.002); CV had a significant decrease during wakefulness after CPAP therapy (*p* < 0.001); and SD1 had a significant decrease during wakefulness after CPAP therapy (*p* = 0.047), but no significant changes during sleep for any of these measures.

In analysis of frequency domain measures, HFP significantly decreased in sleep from 1.78 (95% CI 1.58–2.02) at baseline to 1.61 (95% CI 1.40–1.85) at 3‐month follow‐up (*p* = 0.014). LHR significantly increased in sleep from 3.50 (95% CI 2.94 to 4.18) at baseline to 4.02 (95% CI 3.30 to 4.90) at follow‐up (*p* = 0.014).

In a subgroup of participants with objective CPAP adherence data (*N* = 41), we repeated the pre‐post analysis adjusting for adherence (≥ 4 h/night on ≥ 70% of nights). The direction and magnitude of HRV changes remained consistent, and adjustment for adherence did not materially alter the results (Table S1).

## 4 Discussion

In this study of well‐phenotyped middle‐aged individuals with PAF and moderate to severe SDB, we leveraged a unique opportunity to examine diurnal patterns and the effect of CPAP therapy on indices of autonomic dysfunction. We observe significant inverse associations between SDB severity, defined by the frequency burden of apneic and hypopneic events, and HRV measures during wakefulness; while select associations were also present during sleep (e.g., SD2 and DFA_alpha2), these were fewer and generally less consistent compared to those observed during wakefulness. Nadir SaO_2_, reflecting the degree of hypoxic decline, was associated with sympathovagal imbalance and alterations in time domain HRV in both sleep and wake state. The extent of sleep‐related hypoxia (T90) showed significant inverse associations with time domain measures during wakefulness only. Additionally, the relationships between T90 and other time and frequency domain measures were stronger during wakefulness compared to sleep. Unlike markers of respiratory events and hypoxic dips, T90 was not associated with frequency‐based HRV measures. T90 had consistent relationships with a range of HRV indices, particularly during wakefulness. Therefore, contrary to our a priori hypothesis, these results demonstrate a pattern of stronger associations between SDB severity and sleep‐related hypoxic influences with alterations in biomarkers of autonomic function during wakefulness relative to sleep.

Contrary to our a priori hypothesis that nocturnal associations between SDB and HRV would predominate, we observed stronger relationships during wakefulness. This hypothesis was based on the established pathophysiology of SDB, in which apneic events and desaturations during sleep provoke acute autonomic fluctuations. However, the stronger wakeful associations likely reflect sustained (carry‐over) autonomic effects from repeated nocturnal hypoxic and arousal‐related stimuli. Chronic exposure to these stressors may result in persistent daytime autonomic dysregulation, manifesting as altered HRV even in the absence of acute respiratory events. This pattern is consistent with prior observations of sustained daytime sympathetic activation and blood pressure elevations in OSA, and extends these findings to patients with PAF. Together, these results provide novel insight into the diurnal and chronobiologic impact of SDB on autonomic function, highlighting the importance of daytime autonomic alterations in this population [16].

In response to CPAP, we observed divergent diurnal changes in HRV. During sleep, HFP decreased and LHR increased, while SD2 increased; during wakefulness, time‐domain measures (SDNN, RMSSD, CV, SD1) and SD2 decreased. These changes do not indicate a uniform “restoration of balance”. Although an increased LHR may suggest relative sympathetic dominance, the LF/HF ratio has well‐recognized limitations and does not consistently reflect sympathovagal balance. In particular, LF reflects baroreflex‐mediated and vagal influences, while HF is sensitive to respiratory mechanics; thus, CPAP‐related changes in intrathoracic pressure may alter these components independent of true sympathetic activation. The observed reductions in variability (e.g., SDNN, RMSSD during wakefulness and HFP during sleep) may partly reflect normalization from the heightened variability seen in untreated SDB rather than worsening autonomic function. The opposite directional change in SD2 likely reflects state‐dependent effects, with stabilization during sleep and reduced daytime fluctuations as autonomic burden decreases. These findings were consistent after accounting for CPAP adherence, supporting their robustness. Together, these results highlight the complex, state‐dependent effects of CPAP on autonomic regulation rather than uniform improvement across HRV measures [17].

Our study reveals that autonomic alterations are more pronounced during wakefulness in OSA which is consistent with prior findings [14] of sustained elevations in blood pressure during the daytime, that is, not limited to sleep in patients with obstructive sleep apnea. We now extend these prior observations to the pathophysiology of PAF. These alterations may also be explained by the association between HRV and hypoxia, as well as the severity of SDB [18]. Notably, we identify significant alterations in both time and frequency domain HRV measures after CPAP therapy, which has important implications for the assessment and treatment of hypertension in patients with SDB [19]. Previous studies further support the concept that SDB has sustained effects on autonomic function beyond the sleep period, which likely contributes to cardiovascular disease risk [20]. Our findings have important clinical implications in patients with AF and moderate‐to‐severe sleep apnea. The observed diurnal variations in HRV indicate autonomic dysfunction that could impact outcomes like hypertension, heart failure progression, and cardiovascular events. HRV is a well‐established surrogate marker of autonomic nervous system function, and lower HRV has been associated with increased cardiovascular risk, including arrhythmia burden, hypertension, and mortality. Alterations in HRV measures following CPAP therapy observed in our study may represent early indicators of favorable autonomic remodeling. Although our study did not directly assess long‐term cardiovascular outcomes, prior research suggests that improved autonomic function may predict reduced AF recurrence and cardiovascular events. Optimization of nocturnal autonomic balance through SDB treatment may provide a novel upstream therapy approach to improve AF control and reduce associated morbidity [21, 22, 23, 24].

Additionally, studies have suggested that timed drug administration and chronotherapeutic drug delivery could be valuable approaches for optimizing medication efficacy in the treatment of cardiovascular disease [19, 25]. Our findings suggest potential implications for chronotherapeutics, particularly in the context of managing autonomic dysfunction associated with sleep apnea and AF. Specifically, understanding the circadian rhythms of the ANS in this population could be crucial in informing chronotherapeutic strategies. Such strategies might include the timing of antiarrhythmic drug administration to coincide with periods when the vagal tone is at its peak, thereby maximizing the drugs' efficacy overnight. This approach aligns with the goal of addressing the underlying autonomic irregularities characteristic of sleep apnea and AF more effectively [3, 19, 25]. The sustained effects of SDB on vascular biology beyond the sleep period may contribute to the evolution of systemic hypertension, the latter a recognized risk for AF and may also inform the approach to the timing of antihypertensive medications in those with SDB and AF [14].

Examination of CPAP intervention among participants with moderate to severe SDB (*n* = 55) revealed that the SDNN, LF, HF, and the LHR all decreased after CPAP therapy on the same night [26]. Consistent with our study findings, the overwhelming body of research indicates that restoring the sympathetic balance in patients can lower their chance of developing cardiovascular disease [27]. As noted in previous studies, CPAP therapy resulted in alteration of sympathovagal balance, further demonstrating the impact of SDB treatment on HRV measures.

The absolute or relative power distribution using the HRV can be used to characterize HRV frequency domain metrics. The LHR, for example, is considered a measure of sympathovagal balance. A higher measure is suggestive of sympathovagal system imbalance, as observed in SDB [28], which is an indicator of higher risk for cardiovascular disease and PAF [9]. Frequency distribution between 0.003 and 0.04 Hz, which is connected to temperature regulation, circadian shifts, and other less well‐known processes, is part of the VLF (very low frequency) spectrum [29]. Studies have indicated that patients with SDB have a greater VLF and LF power in HRV [28]. The VLF and LF power spectra are generally associated with the sympathetic nervous system and the neurological control of heart rate [30]. Furthermore, an increase in SDB severity is associated with a rise in VLF indices, which may reflect increased sympathetic nervous system activity [26]. Consistent with these observations, the LHR measures in our current work increased after CPAP treatment, indicating that CPAP therapy influenced autonomic regulation and altered sympathovagal balance in individuals with moderate to severe SDB.

Strengths of the study include its well‐phenotyped sample and prospective investigation of concurrent collection of objective sleep–wake measures via actigraphy and continuous ECG monitoring. We applied rigor to the approach to HRV analysis using standard methodology with pre‐processing and visual inspection of the data, enabling the examination of nearly 50 K epochs of ECG and concurrent sleep and electrophysiologic data with a high degree of completeness (7–24 days) despite a moderate sample size. However, several limitations must be acknowledged. Methodologically, HRV recordings, often considered the gold standard for clinical ANS assessment, are influenced by circadian rhythms, core body temperature, metabolism, the sleep cycle, and the renin‐angiotensin system [9, 30], yet direct measures of autonomic function such as circulating or urinary catecholamines or microneurography assessments were not collected. Additionally, while actigraphy provided objective sleep–wake data, it may not have fully captured daytime naps or fragmented sleep, which could influence diurnal autonomic function patterns. Furthermore, although we accounted for the influence of antiarrhythmic medications in our statistical models, lifestyle factors such as diet, physical activity, AF persistence/duration, and medication adherence were not directly measured, introducing potential residual confounding that may affect the observed associations between SDB, HRV, and CPAP therapy. In addition, because multiple statistical tests were performed and no formal correction for multiple comparisons was applied, the findings should be interpreted as exploratory and hypothesis‐generating.

Future prospective studies incorporating detailed assessments of these confounders could better elucidate their impact and clarify CPAP's independent role in modulating autonomic function. Regarding generalizability, the cohort's predominantly White composition (99.1%) limits the applicability of our findings, as autonomic responses and SDB prevalence may vary across racial groups, potentially altering HRV and CPAP effects in more diverse populations. Additionally, the 3‐month follow‐up period allowed us to detect initial changes in HRV measures post‐CPAP therapy, but this duration may not fully reflect the long‐term autonomic remodeling or cardiovascular benefits of CPAP, which could require 6 months or more to stabilize; future studies with extended follow‐up or integration of longitudinal data could further elucidate CPAP's sustained impact on autonomic function and AF burden. Despite these limitations, the novelty of our study lies in the integration of continuous, multi‐day autonomic monitoring with objective sleep–wake assessment, allowing for detailed characterization of diurnal variations in HRV and the modulatory effect of CPAP therapy in patients with PAF and SDB.

In summary, HRV, as a surrogate of autonomic function, has a significant association with SDB indices in PAF, and this association is stronger during wakefulness. Further, reversal of SDB pathophysiology with CPAP therapy in AF resulted in alterations of sympathovagal balance. Future work to elucidate the long‐term facilitation of autonomic activity is warranted, including designing studies with the ability to draw parallels between hypertension and sleep indices. Given the observational nature of these findings and the nuanced, state‐dependent HRV responses to CPAP, further investigation with long‐term outcome data is needed to validate the clinical impact of HRV changes and explore how the timing of antiarrhythmic and antihypertensive medications may inform chronotherapeutic strategies in SDB and AF.

## Author Contributions

All authors made significant intellectual contributions to the design of the study, data analysis, drafting, and approving the final version of the manuscript. All authors agree to be accountable for all aspects of this work.

## Funding

This work was supported by NHLBI/NIH (SAFEBEAT, NCT02576587).

## Ethics Statement

The study protocol was approved by the Institutional Review Boards of University Hospitals Case Medical Center and the Cleveland Clinic.

## Consent

Written informed consent was obtained from all participants prior to enrollment.

## Conflicts of Interest

The authors have no conflicts to disclose. H.K.W. serves on the advisory board of Resmed.

## Supporting information


**Table S1:** Heart rate variability measures at baseline and post‐CPAP therapy among patients with objective CPAP adherence data, adjusted for adherence and clinical covariates.

## Data Availability

The data generated during this study are available from the corresponding author upon reasonable request.

## References

[1] L. Y. Chen and W. K. Shen , “Epidemiology of Atrial Fibrillation: A Current Perspective,” Heart Rhythm 4, no. 3 Suppl (2007): S1–S6, 10.1016/j.hrthm.2006.12.018. 17336876

[2] M. H. Kim , S. S. Johnston , B. C. Chu , M. R. Dalal , and K. L. Schulman , “Estimation of Total Incremental Health Care Costs in Patients With Atrial Fibrillation in the United States,” Circulation. Cardiovascular Quality and Outcomes 4, no. 3 (2011): 313–320, 10.1161/CIRCOUTCOMES.110.958165. 21540439

[3] P. R. Genta , L. F. Drager , and G. Lorenzi Filho , “Screening for Obstructive Sleep Apnea in Patients With Atrial Fibrillation,” Sleep Medicine Clinics 12, no. 1 (2017): 99–105, 10.1016/j.jsmc.2016.10.009. 28159101

[4] A. M. May , L. Wang , D. H. Kwon , et al., “Sleep Apnea Screening Instrument Evaluation and Novel Model Development and Validation in the Paroxysmal Atrial Fibrillation Population,” International Journal of Cardiology. Heart & Vasculature 31 (2020): 100624, 10.1016/j.ijcha.2020.100624. 33364332 PMC7752750

[5] P. K. Stein and Y. Pu , “Heart Rate Variability, Sleep and Sleep Disorders,” Sleep Medicine Reviews 16, no. 1 (2012): 47–66, 10.1016/j.smrv.2011.02.005. 21658979

[6] L. Zhang , Y. Hou , and S. S. Po , “Obstructive Sleep Apnoea and Atrial Fibrillation,” Arrhythmia & Electrophysiology Review 4 (2015): 14–18. 26835094 10.15420/aer.2015.4.1.14 PMC4711541

[7] D. Linz , A. G. Brooks , A. D. Elliott , et al., “Variability of Sleep Apnea Severity and Risk of Atrial Fibrillation: The VARIOSA‐AF Study,” JACC. Clinical Electrophysiology 5, no. 6 (2019): 692–701, 10.1016/j.jacep.2019.03.005. 31221356

[8] S. A. Sands , R. L. Owens , and A. Malhotra , “New Approaches to Diagnosing Sleep‐Disordered Breathing,” Sleep Medicine Clinics 11, no. 2 (2016): 143–152, 10.1016/j.jsmc.2016.01.005. 27236052 PMC5125379

[9] A. A. Khan , G. Y. H. Lip , and A. Shantsila , “Heart Rate Variability in Atrial Fibrillation: The Balance Between Sympathetic and Parasympathetic Nervous System,” European Journal of Clinical Investigation 49, no. 11 (2019): e13174, 10.1111/eci.13174. 31560809

[10] A. S. Stöberl , E. I. Schwarz , S. R. Haile , et al., “Night‐To‐Night Variability of Obstructive Sleep Apnea,” Journal of Sleep Research 26, no. 6 (2017): 782–788, 10.1111/jsr.12558. 28548301

[11] N. Ahmadi , G. K. Shapiro , S. A. Chung , and C. M. Shapiro , “Clinical Diagnosis of Sleep Apnea Based on Single Night of Polysomnography vs. Two Nights of Polysomnography,” Sleep & Breathing = Schlaf & Atmung 13, no. 3 (2009): 221–226, 10.1007/s11325-008-0234-2. 19067010

[12] L. M. Donovan and V. K. Kapur , “Prevalence and Characteristics of Central Compared to Obstructive Sleep Apnea: Analyses From the Sleep Heart Health Study Cohort,” Sleep 39, no. 7 (2016): 1353–1359, 10.5665/sleep.5962. 27166235 PMC4909617

[13] F. Togo , N. S. Cherniack , and B. H. Natelson , “Electroencephalogram Characteristics of Autonomic Arousals During Sleep in Healthy Men,” Clinical Neurophysiology: Official Journal of the International Federation of Clinical Neurophysiology 117, no. 12 (2006): 2597–2603, 10.1016/j.clinph.2006.07.314. 17011823 PMC2289770

[14] P. Valensi , “Autonomic Nervous System Activity Changes in Patients With Hypertension and Overweight: Role and Therapeutic Implications,” Cardiovascular Diabetology 20, no. 1 (2021): 170, 10.1186/s12933-021-01356-w. 34412646 PMC8375121

[15] M. J. Cowan , “Measurement of Heart Rate Variability,” Western Journal of Nursing Research 17, no. 1 (1995): 32–111, 10.1177/019394599501700104. 7863645

[16] H. Qin , N. Steenbergen , M. Glos , et al., “The Different Facets of Heart Rate Variability in Obstructive Sleep Apnea,” Frontiers in Psychiatry 12 (2021): 642333, 10.3389/fpsyt.2021.642333. 34366907 PMC8339263

[17] G. E. Billman , “The LF/HF Ratio Does Not Accurately Measure Cardiac Sympatho‐Vagal Balance,” Frontiers in Physiology 4 (2013): 26, 10.3389/fphys.2013.00026. 23431279 PMC3576706

[18] I. Szollosi , H. Krum , D. Kaye , and M. T. Naughton , “Sleep Apnea in Heart Failure Increases Heart Rate Variability and Sympathetic Dominance,” Sleep 30, no. 11 (2007): 1509–1514, 10.1093/sleep/30.11.1509. 18041483 PMC2082090

[19] M. H. Smolensky , “Chronobiology and Chronotherapeutics. Applications to Cardiovascular Medicine,” American Journal of Hypertension 9, no. 4 Pt 3 (1996): 11S–21S, 10.1016/0895-7061(95)00405-x. 8722412

[20] D. Raman , F. Kaffashi , L. Y. Lui , et al., “Polysomnographic Heart Rate Variability Indices and Atrial Ectopy Associated With Incident Atrial Fibrillation Risk in Older Community‐Dwelling Men,” JACC. Clinical Electrophysiology 3, no. 5 (2017): 451–460, 10.1016/j.jacep.2016.09.001. 28534047 PMC5437978

[21] T. Klingenheben , G. Grönefeld , Y. G. Li , and S. H. Hohnloser , “Heart Rate Variability to Assess Changes in Cardiac Vagal Modulation Prior to the Onset of Paroxysmal Atrial Fibrillation in Patients With and Without Structural Heart Disease,” Annals of Noninvasive Electrocardiology 4, no. 1 (1999): 19–26, 10.1111/j.1542-474X.1999.tb00360.

[22] C. F. George , T. W. Millar , and M. H. Kryger , “Sleep Apnea and Body Position During Sleep,” Sleep 11, no. 1 (1988): 90–99, 10.1093/sleep/11.1.90. 3363274

[23] N. M. Punjabi , “The Epidemiology of Adult Obstructive Sleep Apnea,” Proceedings of the American Thoracic Society 5 (2008): 136–143, 10.1513/pats.200709-155MG. 18250205 PMC2645248

[24] F. G. de Oliveira , I. Pinto , B. Valdigem , T. Senra , and A. Bertolami , “Evaluation of Late Atrial Enhancement by Cardiac Magnetic Resonance Imaging in Patients With Obstructive Sleep Apnea,” Sleep Medicine 74 (2020): 204–210, 10.1016/j.sleep.2020.06.026. 32861012

[25] L. Zhang and M. K. Jain , “Circadian Regulation of Cardiac Metabolism,” Journal of Clinical Investigation 131, no. 15 (2021): e148276, 10.1172/JCI148276. 34338224 PMC8321567

[26] N. Efazati , B. Rahimi , M. Mirdamadi , M. Edalatifard , and A. Tavoosi , “Changes in Heart Rate Variability (HRV) in Patients With Severe and Moderate Obstructive Sleep Apnea Before and After Acute CPAP Therapy During Nocturnal Polysomnography,” Sleep Science (Sao Paulo, Brazil) 13, no. 2 (2020): 97–102, 10.5935/1984-0063.20200003. 32742578 PMC7384525

[27] D. H. Park , C. J. Shin , S. C. Hong , et al., “Correlation Between the Severity of Obstructive Sleep Apnea and Heart Rate Variability Indices,” Journal of Korean Medical Science 23, no. 2 (2008): 226–231, 10.3346/jkms.2008.23.2.226. 18437004 PMC2526439

[28] Y. S. Kim , S. Y. Kim , D. Y. Park , H. W. Wu , G. S. Hwang , and H. J. Kim , “Clinical Implication of Heart Rate Variability in Obstructive Sleep Apnea Syndrome Patients,” Journal of Craniofacial Surgery 26, no. 5 (2015): 1592–1595, 10.1097/SCS.0000000000001782. 26114507

[29] G. G. Berntson , J. T. Bigger, Jr. , D. L. Eckberg , et al., “Heart Rate Variability: Origins, Methods, and Interpretive Caveats,” Psychophysiology 34, no. 6 (1997): 623–648, 10.1111/j.1469-8986.1997.tb02140.x. 9401419

[30] F. Shaffer and J. P. Ginsberg , “An Overview of Heart Rate Variability Metrics and Norms,” Frontiers in Public Health 5 (2017): 258, 10.3389/fpubh.2017.00258. 29034226 PMC5624990

