# Supplementary material for: Sleep Apnea and Paroxysmal Atrial Fibrillation: Diurnal Patterning of Autonomic Dysfunction and Influence of CPAP Therapy
Source: J Arrhythm. 2026 Jun 10;42(3):e70352. doi: 10.1002/joa3.70352 (PMC13251432; doi:10.1002/joa3.70352)
Supplement: Supplementary file 1 — Table S1: Heart rate variability measures at baseline and post‐CPAP therapy among patients with objective CPAP adherence data, adjusted for adherence and clinical covariates. [file JOA3-42-e70352-s001.docx]

| **Supplemental Table S1.** Heart rate variability measures at baseline and post-CPAP therapy among patients with objective CPAP adherence data, adjusted for adherence and clinical covariates. | | | | | | |
| --- | --- | --- | --- | --- | --- | --- |
| **Variable** | **Sleep** | | | **Awake** | | |
|  | **Baseline** | **Follow-up** |  | **Baseline** | **Follow-up** |  |
|  | **LSM (95%CI)** | **LSM (95%CI)** | **P value** | **LSM (95%CI)** | **LSM (95%CI)** | **P value** |
| MNN | 0.895 (0.823,0.966) | 0.917 (0.846,0.988) | ***0.010*** | 0.777 (0.706,0.848) | 0.804 (0.732,0.875) | ***0.002*** |
| SDNN | 0.030 (0.024,0.036) | 0.030 (0.024,0.037) | 0.80 | 0.031 (0.025,0.037) | 0.029 (0.023,0.035) | ***0.010*** |
| RMSSD | 0.030 (0.022,0.038) | 0.028 (0.020,0.036) | ***0.042*** | 0.026 (0.018,0.034) | 0.024 (0.016,0.032) | ***0.038*** |
| CV* | 0.030 (0.026,0.036) | 0.031 (0.026,0.036) | 0.78 | 0.036 (0.030,0.042) | 0.034 (0.029,0.040) | ***0.032*** |
| SD1* | 0.017 (0.012,0.022) | 0.016 (0.012,0.022) | 0.53 | 0.013 (0.010,0.018) | 0.013 (0.010,0.018) | 0.50 |
| SD2* | 0.033 (0.027,0.039) | 0.034 (0.028,0.041) | 0.065 | 0.035 (0.029,0.042) | 0.034 (0.028,0.041) | 0.32 |
| SDRatio* | 0.527 (0.449,0.619) | 0.496 (0.422,0.583) | ***0.043*** | 0.404 (0.344,0.475) | 0.404 (0.343,0.475) | 0.96 |
| LFP* | 4.65 (3.82,5.66) | 4.81 (3.95,5.85) | 0.29 | 5.01 (4.11,6.09) | 5.11 (4.19,6.23) | 0.48 |
| HFP* | 1.99 (1.55,2.54) | 1.77 (1.38,2.27) | ***0.016*** | 1.14 (0.891,1.46) | 1.16 (0.907,1.49) | 0.66 |
| LHR* | 3.03 (2.02,4.54) | 3.56 (2.37,5.34) | ***0.012*** | 5.29 (3.53,7.93) | 5.36 (3.57,8.06) | 0.83 |
| DFA_Alpha1 | 0.973 (0.910,1.035) | 0.990 (0.926,1.053) | 0.16 | 1.066 (1.003,1.13) | 1.072 (1.008,1.13) | 0.62 |
| DFA_Alpha2 | 0.645 (0.590,0.701) | 0.633 (0.577,0.690) | 0.41 | 0.823 (0.767,0.878) | 0.800 (0.744,0.857) | 0.11 |
